# Advances in the Biosynthesis of Terpenoids and Their Ecological Functions in Plant Resistance

**DOI:** 10.3390/ijms241411561

**Published:** 2023-07-17

**Authors:** Changyan Li, Wenjun Zha, Wei Li, Jianyu Wang, Aiqing You

**Affiliations:** 1 Food Crops Institute, Hubei Academy of Agricultural Sciences, Hubei Key Laboratory of Food Crop Germplasm and Genetic Improvement, Laboratory of Crop Molecular Breeding, Ministry of Agriculture and Rural Affairs, Wuhan 430064, China; zwj_19850202@hbaas.com (W.Z.); 202221107012051@stu.hubu.edu.cn (J.W.); 2 College of Life Science and Technology, Huazhong Agricultural University, Wuhan 430070, China; liwei199106@zju.edu.cn; 3 School of Life Sciences, Hubei University, Wuhan 430062, China; 4 Hubei Hongshan Laboratory, Wuhan 430070, China

**Keywords:** terpenoids, insects, disease, plant resistance, interaction

## Abstract

Secondary metabolism plays an important role in the adaptation of plants to their environments, particularly by mediating bio-interactions and protecting plants from herbivores, insects, and pathogens. Terpenoids form the largest group of plant secondary metabolites, and their biosynthesis and regulation are extremely complicated. Terpenoids are key players in the interactions and defense reactions between plants, microorganisms, and animals. Terpene compounds are of great significance both to plants themselves and the ecological environment. On the one hand, while protecting plants themselves, they can also have an impact on the environment, thereby affecting the evolution of plant communities and even ecosystems. On the other hand, their economic value is gradually becoming clear in various aspects of human life; their potential is enormous, and they have broad application prospects. Therefore, research on terpenoids is crucial for plants, especially crops. This review paper is mainly focused on the following six aspects: plant terpenes (especially terpene volatiles and plant defense); their ecological functions; their biosynthesis and transport; related synthesis genes and their regulation; terpene homologues; and research and application prospects. We will provide readers with a systematic introduction to terpenoids covering the above aspects.

## 1. Introduction

Crops are one of the most important energy sources for humans. With the continuous growth of the world’s population, there is an urgent need to improve the output and quality of food through various means to meet societal needs. Insect pests reduce crop production by approximately 10% per year [1]. At present, we mainly rely on chemical pesticides to control pests in grain production; however, the use of a large number of pesticides is bound to have many adverse effects on our lives, including greater pollution, poorer food quality, and increasingly resistant pests.

With advances in science and technology, the introduction of exogenous resistance genes into crops through plant genetic engineering has shown good potential for agricultural pest control [1]. For example, in the most widely used Bt transgenic crops, the Bt protein has the ability to specifically kill target pests. Hence, introducing this gene into crops through plant transgenic technology can provide them with some resistance against pests [2,3,4,5,6]. However, given the increasing prevalence of insect pests resistant to the Bt protein in recent years, other methods need to be developed as candidates for pest control.

Terpenoids are the most abundant secondary metabolites in plants, comprising approximately 25,000 kinds of compounds. Structurally, terpenes are composed of single or multiple five-carbon units which can be divided into sesquiterpenes, monoterpenes, sesquiterpenes, diterpenes, triterpenes, tetraterpenes, and high polyterpenes, according to the number of five-carbon units. Monoterpenes and sesquiterpenes are important components of the volatile compounds produced by plants, and they play an important role in the interactions and defense reactions between plants, microorganisms, and animals. Thirty years ago, researchers discovered that plants have unique defense mechanisms against pests. Plants can release volatiles after being eaten by pests, and these volatiles attract the natural enemies of the pests to achieve pest control [7,8]. Following the discovery of this unique defense mode in plants, the “regulation” strategy of using the tritrophic interaction of “crop–pest–natural enemy” to control pests came into being in the process of crop production. According to this concept, pesticides were first used to “kill” pests in a large area; subsequently, small amounts of pesticides were used to “kill” pests in combination with the corresponding “regulation” strategies for controlling pests. In theory, pests will not overcome the indirect defense strategy of attracting natural enemies, and this is a sustainable anti-insect strategy.

## 2. Plant Defense and Terpene Volatiles

There are millions of insects on earth, most of which feed on plants, and plants in turn have evolved efficient and sophisticated defense mechanisms to reduce the harm caused by herbivorous insects [9,10]. There are four main mechanisms of defense, and these are summarized as follows.

**Constitutive defense:** Constitutive defense refers to the resistance inherent in plants and it is accompanied by a lifetime of resistance. The advantage of this method is that plants accumulate the corresponding defense substances or form the corresponding defensive morphological structures (such as wax, leaf color, or surface hair) before they are damaged by pests. However, a disadvantage of this defense is that it may waste nutrients and energy that could otherwise be used for growth, development, and reproduction.

**Induced defense:** Induced defense is a mechanism according to which a defensive response is induced in plants by adverse external factors, such as a pest invasion or mechanical damage, which can minimize the energy and substances consumed in plant defense [11]. Induced defenses can be divided into direct and indirect defenses according to the different chemical substances produced by the plants.

**Direct defense:** Direct defense refers to the production of chemical substances that impose nutritional restrictions or toxic effects on pests after they have attacked a plant, thereby affecting the feeding, growth, development, or reproduction of herbivorous insects. During the direct defense process, plants produce substances to repel pests, such as (E)-β-farnesene (which affects cotton aphids (*Aphis gossypii*)), protease inhibitors, and other substances that affect the pests’ digestion and absorption. Toxic compounds such as alkaloids directly kill pests [12,13,14].

**Indirect defense:** Indirect defense is a supplement to direct defense. In this process, when plants are damaged by herbivorous insects, they emit terpene-based volatile organic compounds to attract the enemies of natural pest [15]. For example, linalool and caryophyllene can attract rice lice wasps (*Anagrus nilaparvatae*) to prevent damage from brown planthoppers (*Nilaparvata lugens* (Stal)) [16], and caryophyllene can also attract the parasitic wasp *Cotesia sesamiae*, the natural enemy of corn borer (*Chilo partellus*) larvae, to defend against corn borers [17].

Volatile organic compounds (VOCs) play an important role in plant defense responses. Tens of thousands of VOCs have been identified in plant species, most of which can be divided into three categories: fatty acid derivatives, benzene ring/phenylpropane compounds, and terpenoids [18].

### 2.1. Fatty Acid Derivatives

Plants produce fatty acid derivatives via the lipoxygenase (LOX) pathway. This pathway uses linoleic or linolenic acid as the starting point through the action of lipoxygenase [18,19]. First, a series of 9-hydroperoxidation or 13-hydroperoxidation intermediates are synthesized; these intermediates are then catalyzed by oxidative decarboxylation to produce 6-carbon or 9-carbon alcohols, aldehydes, and their esters (such as (E)-2-hexenal and (Z)-3-hexenol) [20]. Because of their typical green leaf odor, they are also called green leaf volatiles [21]. Intermediate 13-hydroperoxidation can produce jasmonic acid (JA) and methyl jasmonate (MeJA). Green leaf volatiles can be released within minutes of plants being subjected to biotic or abiotic stresses, such as diseases and attacks by insect pests. Fatty acid derivatives may be transmitted to other plants as signals to warn them to take precautions in advance [21,22]. For example, after injury, plants release MeJA into the air and spread it to nearby undamaged plants as a warning of imminent harm [23].

### 2.2. Benzene Ring/Phenylpropane Compounds

Benzene ring/phenylpropane compounds are mainly synthesized via the shikimic acid pathway [24]. Methyl salicylate is one of the most widely studied VOCs among benzene-ring/phenylpropane compounds. To the best of our knowledge, this is the first study to demonstrate the involvement of signal transmission in healthy and diseased plants. Tobacco (*Nicotiana tabacum*) can release methyl salicylate and thereby affect the expression of defense genes in neighboring healthy plants when infected with the tobacco mosaic virus [25]. Many other benzene ring/phenylpropane volatiles are also the main components of flower volatiles, and these compounds may be involved in attracting pollinating insects, such as when snapdragon (*Antirrhinum majus*) plants, which are pollinated by honeybees, emit methyl benzoate during the day when bees are most active, thus attracting bees and encouraging pollination [26].

### 2.3. Terpenoids

Terpenoids are the most abundant VOCs in plants, and more than 80,000 species have been found to date [27]. They are mainly produced via isoprene-like pathways, and isoprene (C5) is the basic unit of which are composed. There are two main pathways for isoprene synthesis in plants: the methyl erythritol-4-phosphate (MEP) pathway located in plastids, which produces monoterpenes (linalool, myrcene, and limonene), diterpenes (geranyl linalool), and their derivatives (TMTT and-ionone), and the mevaleric acid (MVA) pathway, which produces sesquiterpenes (such as (E)-β-farnesene, α-humulene, caryophyllene, and nerolidol) and their derivatives (such as DMNT). Terpenoids are the most diverse functional substances among plant secondary metabolites, and functional studies of terpenoids and their metabolic pathways have become new research hotspots.

## 3. Ecological Functions of Terpenoids

**Figure 1:**
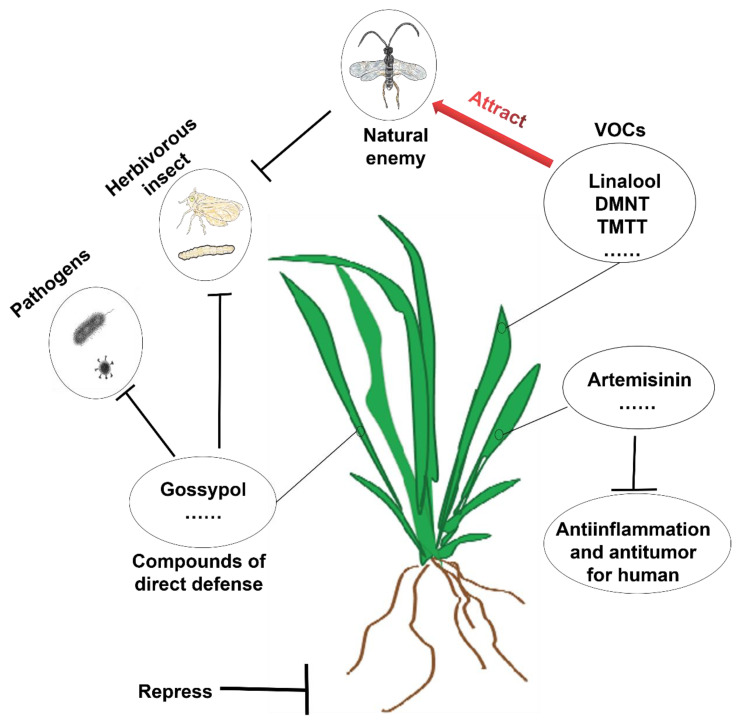
The functions of plant terpenoids and terpene homologues. Terpenoids and terpene homologues play important roles in plants by regulating resistance to biotic stress (including insect and disease resistance). Terpenoids also have many significant impacts on human life, and they can play a role in anti-inflammatory, antioxidant, anti-aggregation, anticoagulant, anti-tumor, sedative, and analgesic activities. Artemisinin is one such example.

Terpenoids play an important role in plant photosynthesis by regulating plant growth and development, pollination, and resistance to external biotic or abiotic stresses [28]. For instance, the tetraterpene carotene can absorb and transmit light energy, and some important hormones in plants (abscisic acid, brassinolide, and gibberellin) are terpene derivatives that can affect plant growth and development. The linalool and nerol produced by tea plants under low temperatures can improve the cold tolerance of neighboring plants [29]. Linalool, (S)-limonene, (E)-nerolidol, and terpinene inhibit *Xanthomonas oryzae* activity [29,30,31,32,33,34]. Limonene and elemene perform antibacterial activities against *Magnaporthe grisea*, and some terpenoids can attract insects for pollination because of their fragrance [35,36]. Terpenoids can also participate in the induced defense responses of plants. For example, (E)-β-farnesene repels aphids, and caryophyllene and linalool attract rice lice wasps [37,38,39,40] (Figure 1).

In addition, terpenoids affect human life in a number of ways, and they are widely used in food processing, perfumes, medicine, biofuels, and other fields. Many volatile terpenoids are fragrant compounds. Limonene, linalool, and nerolidol have unique fragrances and can be used as food additives and perfumes [41,42]. In recent years, several studies have found that all kinds of terpenoids and their glycoside derivatives play various roles in anti-inflammatory, antioxidant, anti-aggregation, anticoagulant, anti-tumor, sedative, and analgesic activities [43,44]. For example, the diterpenoid taxol has anticancer properties, and the sesquiterpene lactone artemisinin has antimalarial functions [45]. Because of their low hygroscopicity, high energy density, and good fluidity at low temperatures, some terpenes have great potential as renewable biofuels [46]. For example, monoterpenes can be used as biogas analogs, while sesquiterpenes and diterpenes can be used as biodiesels [47] (Figure 1).

## 4. Biosynthesis of Terpenoids

Although many types of terpenoids exist, they have similar synthetic pathways. The terpenoid biosynthesis pathway is divided into three steps.

### 4.1. C5 Precursor IPP and DMAPP Formation Phase

**Figure 2:**
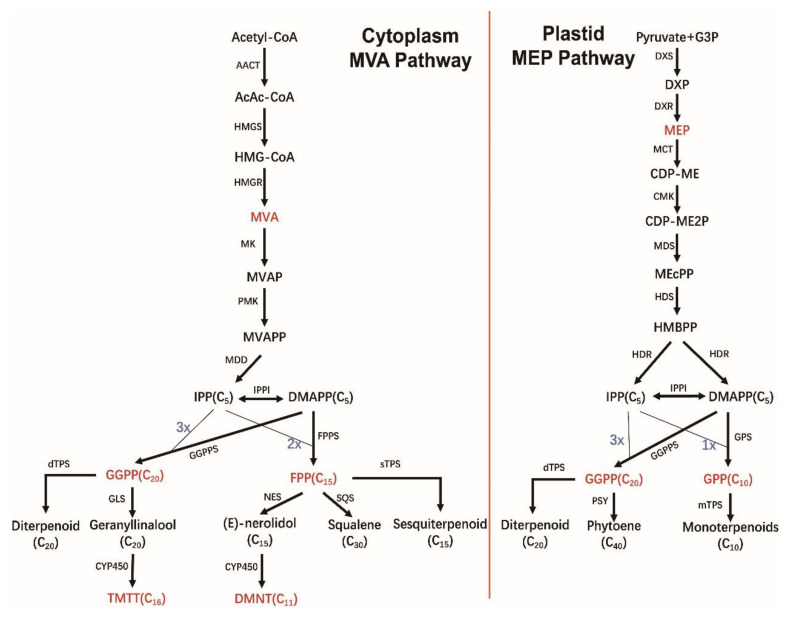
Sketch map of biosynthesis of terpenoids and terpene homologues in plants: the MVA pathway in the cytoplasm and the MEP pathway in plastids. Abbreviations: AACT, acetoacetyl-CoA thiolase; AcAc-CoA, acetoacetyl-CoA; HMGS, HMG-CoA synthase; HMG-CoA, 3-hydroxy-3-methylglutaryl-Coenzyme A; HMGR, HMG-CoA reductase; MVA, mevalonic acid; MK, mevalonate kinase; MVAP, mevalonate 5-phosphate; PMK, phosphomevalonate kinase; MVAPP, mevalonate 5-diphosphate; MDD, mevalonate diphosphate decarboxylase; IPP, isopentenyl diphosphate; IPPI, IPP isomerase; DMAPP, dimethylallyl diphosphate; G3P, glyceraldehyde-3-phosphate; DXS, 1-deoxy-D-xylulose-5-phosphate synthase; DXP, 1-deoxy-D-xylulose-5-phosphate; DXR, 1-deoxy-D-xylulose-5-phosphate reductoisomerase; MEP, 2-C-methylerythritol 4-phosphate; MCT, 2-C-methyl-D-erythritol 4-phosphate cytidylyltransferase; CDP-ME, 4-(cytidine 5’-diphospho)-2-C-methylD-erythritol; CMK, CDP-ME kinase; CDP-ME2P, 4-(cytidine 5’-diphospho)-2-C-methyl-D-erythritol phosphate; MDS, 2-C-methyl-D-erythritol 2,4-cyclodiphosphate synthase; MEcPP, 2-C-methyl-D-erythritol 2,4-cyclodiphosphate; HDS, (E)-4-hydroxy-3-methylbut-2-enyl diphosphate synthase; HMBPP, (E)-4-hydroxy-3-methylbut-2-enyl diphosphate; HDR, (E)-4-hydroxy-3-methylbut-2-enyl diphosphate reductase; GGPPS, geranyl Mapp diphosphate synthase; FPPS, farnesyl diphosphate synthase; GPS, GPP synthase; GGPP, geranylgeranyl diphosphate; FPP, farnesyl pyrophosphate; GPP, geranylvdiphosphate; mTPS, monoterpenoid synthase; sTPS, sesquiterpene synthase; dTPS, diterpene synthases; NES, nerolidol synthase; GLS, geranyllinalool synthase; SQS, squalene synthase; PSY, Phytoene synthase; DMNT, 4,8-dimethylnona-1,3,7-triene; TMTT, 4,8,12-trimethyltrideca-1,3,7,11-tetraene.

The C5 precursor consists of two isomers, IPP and DMAPP, which are mainly synthesized from MVA and MEP. The MVA pathway includes six steps of enzymatic reactions, providing precursors for sesquiterpenes, phytosterols, and triterpenoids such as brassinolide and ubiquinones in the mitochondria. The MEP pathway consists of seven enzymatic steps that mainly act as substrate sources for monoterpenes, diterpenes, carotenoids, and their decomposition products (cytokinins, gibberellins, chlorophyll, tocopherols, and plastids) [48] (Figure 2).

### 4.2. Direct Precursor Formation Stage

Geranyl pyrophosphate (GPP, C10), farnesyl pyrophosphate (FPP, C15), and geranylgeranyl pyrophosphate (GGPP, C20) are the main direct precursors of most terpenoids. GPP is synthesized by the action of GPP synthase (GPS), which is a precursor in monoterpene synthesis [49]. GPS exists in two forms: a homodimer composed of two identical subunits, and a heterodimer composed of a small and a large subunit. FPP is a precursor for the synthesis of sesquiterpenes, triterpenes, and sterols, which are formed by the condensation of one DMAPP molecule and two IPP molecules. GGPP is a precursor of many important substances in plants, including chlorophyll, carotenoids, gibberellins, and tocopherols. It is produced from three IPP molecules and one DMAPP molecule under the action of bovine pyrophosphate synthase (GGPS). Interestingly, GGPS can also act as a large subunit of the GPS heterodimer [49,50].

### 4.3. Terpene Formation and Modification Stage

There have been many studies on the formation stages of IPP, DMAPP, and their direct precursors, and all terpenoids must undergo these two stages. There are few studies on the third stage, especially on the modification processes of terpenoids, yet this stage is the main factor in the enrichment of terpenoid diversity. Most terpenes are catalyzed by terpene synthase (TPS), which removes the bisphosphate groups of the direct precursors GPP, FPP, and GGPP to form monoterpenes (C10), sesquiterpenes (C15), diterpenes (C20), and polyterpene skeletons. Squalene synthase (SQS/farnesyl diphosphate farnesyltransferase) and phytene synthetase (PSY/geranylgeranyl diphosphate gerany ltransferase) can also directly condense two FPP or GGPP molecules to form the sterol precursor squalene (C30) and the carotenoid precursor octahydrolycopene (C40) [51].

After terpenoid carbon skeleton synthesis, terpenoids can be further modified by redox reactions, methylation, acylation, and glycosylation by cytochrome P450 (CYP) and other modifying enzymes, thus providing terpenoids with various structures, complex chemical properties, and unique functional characteristics [52]. The main function of CYP is to oxidize terpenes. For example, in plants, (E,E)-geranyllinalool and (E)-nerolidol can produce the terpene homologues TMTT and DMNT, respectively, under the action of P450 enzymes encoded by AtCYP82G1, and these two substances produce important anti-insect effects in many plants. The specific metabolic responses of terpenes in plants are shown in Figure 2.

### 4.4. Transport of Terpenoids

Few studies have been conducted on the transport and emission of terpenoids in plants after their synthesis. In recent years, following in-depth studies of terpenoid metabolic pathways, an increasing number of researchers have begun to study terpenoid transport. They have found that terpenoid transport is related to ABC transporters. For example, in the leaves of *Vinca minor*, VmABCG1, a member of the superfamily of ABC transporters, is involved in the emission of the nonvolatile monoterpene indole alkaloid vinblastine [53]. In addition, in the hairs of *Artemisia annua*, sesquiterpene (E)-caryophyllene transport is completed under the action of AaPDR3, a member of the ABC transporter superfamily [54]. Capsidiol emissions from *Nicotiana benthamiana* are related to NbABCG1 and NbABCG2 [55]. However, the substrate specificity of these transporters and the reversibility of their transport directions are unclear and require further study.

## 5. Synthetic Genes Related to Terpenoids and Their Transcriptional Regulation

### 5.1. Terpene Synthase (TPS)

Terpene synthase catalyzes the formation of terpenoids, and the abundance of its species is closely related to the presence of a large number of terpene synthase genes in plants [56]. The TPS gene family is a medium-sized family in plants. Except for the bryophyte *Physcomitrella patens*, which contains only one functional TPS gene, plant genomes contain approximately 20–152 TPS genes, some of which have lost their functions during evolution [57].

Based on differences in amino acid sequences, the TPS gene family can be divided into seven subfamilies: TPS-a, TPS-b, TPS-c, TPS-d, TPS-e/f, TPS-g, and TPS-h [58,59]. TPS-a, TPS-b, and TPS-d are mainly involved in plant secondary metabolism. TPS-a is divided into TPS-a1 and TPS-a2, which are involved in sesquiterpene synthesis in dicotyledons and monocotyledons, respectively. TPS-b mainly synthesizes monoterpenes in angiosperms, but almost all of the genes in this subfamily come from dicotyledons (except sorghum). The TPS-c and TPS-e branches contain all the enzymes involved in gibberellin biosynthesis in gymnosperms and angiosperms, as well as an unknown functional terpene synthase containing the DDXXD domain found in Moellendorf’s spikemoss. The TPS-f branches are primarily involved in monoterpene synthesis in dicotyledons. Because the TPS-e branch is believed to be derived from the TPS-f subfamily, they are collectively referred to as the TPS-e/f subfamily. The TPS-g subfamily is closely related to the TPS-b subfamily and participates in the synthesis of acyclic monoterpenes, sesquiterpenes, and diterpenes, while the TPS-h subfamily exists only in gymnosperms and has not been found to synthesize bifunctional diterpenes in angiosperms [57,60].

Based on their structures and catalytic mechanisms, terpene synthases can be divided into two categories (Class I and Class II). Class I consists of ionization-dependent terpene synthases, and the loss of pyrophosphate groups on the substrate depends on the synergism of metal ions such as Mg^2+^ and Mn^2+^. Most monoterpene, sesquiterpene, and diterpene synthases belong to Class I. There are two main conserved domains in the Class I structure: one is the aspartic acid residue-rich domain DDxxD, located at the C-terminus, and the other is the (L, V) (V, L, A) − (N, D) D(L, I, V) × (S, T) xxxE domain, which is known as the NSE/DTE domain. The primary role of these two conserved domains is to coordinate the binding of metal ions and substrate phosphate groups to promote the formation of carbon-positive ion intermediates. Due to the random rearrangement of bonds after the formation of carbon-positive ion intermediates, this type of terpene synthase reacts with a single substrate to produce a variety of products [56]. Class II enzymes are proton-dependent terpene synthases. The substrate produces carbocation intermediates through protonation and catalyzes skeleton rearrangement, which mainly involves diterpene synthase and triterpene synthase [27,61]. A DxDD-conserved domain exists in the Class II structure. The DDxxD domain is ubiquitous in terpene synthases, but some copalyl diphosphates have only the DxxD domain and the natural DDxxD variation sequence NDxxD [62]. Interestingly, a few terpene synthases have both the DDxxD and DxxD domains.

In general, terpene synthase has the following characteristics: (1) It reacts with the same substrate to produce different products, such as OsTPS31 (LOC_Os08g07100), which can react with FPP to produce 14 sesquiterpenes, mainly gingerene and β-sesquiphellandrene [63]. (2) It has the characteristics of a multi-functional enzyme; that is, it can catalyze different substrates (GPP, FPP, and GGPP) to produce different terpenoids, such as OsTPS3 in rice, PlTPS3 in lima beans, and CsLIS/NES in tea plants, all of which have the bifunctional enzyme linalool/nerolidol [63,64,65]. There are even linalool/nerolidol/geranyllinalool synthases in some plants, such as ZmTPS2 in corn, and PlTPS2 and PlTPS4 in lima beans [64,66,67]. (3) The expression of terpene synthase is specific to different tissues and times and can be further induced by biotic or abiotic stresses. For example, some terpene synthase genes are expressed only in specific plant tissues during a specific growth period or are induced after stress [68,69].

### 5.2. Transcriptional Regulation of Terpenoids

Terpenoids play vital roles in plants and are mostly secondary metabolites. These terpenoids have no effect on the growth and development of plants, and their functions are mainly reflected in their ability to protect against various biological and abiotic stresses. Plants are not always in a state of stress; therefore, the synthesis of too many secondary terpenoids may cause waste or even harm to the plant. The synthesis of chemicals in plants involves an investment of energy and resources. If the synthesis of a substance benefits the plant, the plant may maintain the ability; however, if the benefit is not worth the energy cost, the synthesis of a substance will be gradually eliminated [70]. Therefore, during plant evolution, a series of fine regulatory processes have developed around the biosynthesis and metabolism of specific terpenoids that enable the inducement of their synthesis in specific tissues or developmental periods, as well as under various biotic and abiotic stresses. In this way, while plants are kept resistant to external adverse environmental stresses, energy consumption is also reduced, resulting in a “win-win” situation. This process is mainly regulated by transcription factors at the gene transcriptional level.

Transcription factors are DNA-binding proteins that recognize and bind to specific *cis*-elements in target gene promoters [71]. Transcription factors can be divided into different families based on differences in their DNA-binding domains. There are at least 64 transcription factor families in the vascular plant genome [72]. Previous studies have shown that transcription factor families such as WRKY, MYB, bHLH, AP2/ERF, NAC, bZIP, SPL, and YABBY are involved in the metabolic pathways of specific terpenoids in plants (Table 1).

The WRKY transcription factor is involved in the synthesis of triterpene in cotton, sesquiterpene artemisinin in *Artemisia annua*, diterpene antitoxin in rice, taxol in *Taxus chinensis*, tanshinone in *Salvia miltiorrhiza*, and the triterpenoid compound ginsenoside in North American ginseng (*Panax quinquefolius*) by binding to the W-box element [75,80,84,87,89,90,94]. MYB transcription factors promote or inhibit terpenoid synthesis. For example, in spearmint (*Mentha spicata*), MsMYB reduces the downstream terpene content by inhibiting the expression of geranyl diphosphate synthase, whereas in *Salvia miltiorrhiza*, SmMYB36 promotes tanshinone biosynthesis [82,91]. BHLH transcription factors regulate monoterpene, sesquiterpene, and triterpene derivatives. For example, in butterfly orchids (*Phalaenopsis*), bHLH4 and bHLH6 promote the accumulation of monoterpenes and enhance their aroma [88]. In *Arabidopsis thaliana*, MYC2 (bHLH) can promote the production of sesquiterpene (E)-β-caryophyllene, and the TSAR1/2 (bHLH) transcription factor in alfalfa (*Medicago truncatula*) can regulate the synthesis of triterpenoid saponins [74,81]. The AP2/ERF transcription factors are involved in the biosynthesis of monoterpenes, sesquiterpenes, and diterpene derivatives. For example, the CitERF71 in citrus plants regulates geraniol production by controlling the expression of *CitTPS16*. The AaERF1 and AaERF2 in *Artemisia annua* and the ZmEREB58 in maize regulate sesquiterpene synthesis, whereas the SmERF128 in *Salvia miltiorrhiza* regulates tanshinone synthesis [76,79,92,95]. Few studies have examined the regulation of terpenoids by NAC transcription factors, which may be involved in the synthesis of monoterpenes and carotenoids [73,93]. bZIP transcription factors involved in the synthesis of diterpene derivatives and artemisinin, such as rice OsTGAP1, promote plant protegrin synthesis [85]. The inhibition of the rice OsTGAP1 interaction protein OsbZIP79 inhibits its synthesis, and the bZIP1 in *Artemisia annua* can bind to the ABRE element (ABA-responsive element) on the ADS and CYP71AV1 gene promoters to promote artemisinin synthesis [77,86]. SPL transcription factors regulate the biosynthesis of sesquiterpenes and artemisinin. For example, the AaSPL2 in *Artemisia annua* promotes artemisinin accumulation [78]. The YABBY transcription factor MsYABBY5 can regulate the production of monoterpenes in spearmint [83].

To date, few studies have been conducted on the transcriptional regulation of terpenoid metabolic pathways, and those that have been carried out have mainly focused on only a few terpenoids. A further understanding of the regulation of terpenoids is needed to provide a strong guarantee for increasing the yields of target terpenoids in plants [96].

## 6. Progress of Terpene Homologue Research

### 6.1. Discovery and Ecological Function of Terpene Homologues DMNT and TMTT

These two substances were first identified as new chemicals when they were isolated from the essential oil of cardamom (*Eletteria cardamomum*) [97] which, because of its flavor-enhancing properties, it is used as an additive in perfumes and food.

The ecological functions of DMNT and TMTT were first discovered in 1990. Lima bean (*Phaseolus lunatus*) plants can emit DMNT and TMTT after being attacked by mites and can strongly attract the natural enemies of female phytoseiid mites (*Phytoseiulus persimilis*), which are from Chile [7]. DMNT and TMTT were also detected in maize seedling metabolites in the same year; they can effectively lure female *Cotesia marginiventris* parasitoids to their natural prey, the pest *Parasa consocia* (*Latoia consocia*) [7]. Consequently, researchers have conducted a series of ecological studies on these two substances.

#### 6.1.1. Attracting Pollination Insects

Terpene homologues are also found in the aromatic components released by some plants, and they can help plants to attract pollinating insects [98]. For example, the TMTT released by African orchids (*Aeranis friesiorum*) can attract moths for pollination [99]. Among the flower aroma components of some yucca (*Yucca smalliana*) varieties, DMNT is the most abundant substance, and it plays a role in attracting moths for pollination [100,101]. DMNT and TMTT play important roles in attracting insect pollinators; however, the attraction mechanism remains unclear.

#### 6.1.2. Attracting the Natural Enemies of Insects 

Since the discovery of terpene homologues, a large number of studies have found that the release of DMNT and TMTT from most plants is very low under normal conditions, and that their release increases significantly only when such an increase is induced by biotic or abiotic stress (especially by pests). DMNT and TMTT can attract the natural enemies of insect pests and thereby reduce the damage caused by these pests. For instance, cotton (*Gossypium* spp.), when attacked by chewing caterpillars or sucking worms, can release DMNT and TMTT and strongly attract natural enemies such as female parasitoids [102]. Terpene homologues commonly attract the natural enemies of insect pests in higher plants. In some cases, this may be due to the combined action of many substances (Figure 2).

#### 6.1.3. Pest Avoidance

Terpene homologues tend to keep away some pests. For example, cotton treated with JA can induce the production of TMTT and have a repellent effect on the cotton aphid [103].

#### 6.1.4. Inducing Defense Responses in Neighboring Plants

Terpene homologues can also induce defense responses in adjacent healthy plants. This phenomenon was first reported in lima bean plants. Feeding *Tetranychus urticae* with lima bean plants induced not only the production of terpene homologues, but also the expression of resistance genes such as PR-2 and lipoxygenase LOX in uninjured neighboring plants, improving their resistance to *Tetranychus urticae* [104,105]. A similar phenomenon was observed in tea plants. When pests feed on tea plants, DMNT can induce the upregulation of the LOX1 and LOX3 genes in healthy adjacent tea plants, thus increasing their JA content and improving their resistance to pests [106]. However, the specific mechanism by which volatiles induce resistance in neighboring plants remains unclear.

Many studies have been conducted on the use of DMNT and TMTT to attract the natural enemies of insect pests, but there is an urgent need to strengthen the research on the relationship between DMNT and pollinating insects, adjacent plants, and corresponding pests to further explore the ecological functions of DMNT and TMTT.

### 6.2. Biosynthesis of DMNT and TMTT

Since the discovery of DMNT and TMTT, scientists have explored their metabolic pathways in plants. Isotope signals were detected in DMNT and TMTT when deuterium-labeled nerolidol and geranyl linalool were added to lima bean leaves, indicating that the biosynthesis of DMNT and TMTT may be triggered by the oxidative degradation of nerolidol and geranyllinalool [107,108]. Subsequent studies have found that the synthesis of DMNT and TMTT in plant leaves is divided into two steps: first, the formation of the tertiary alcohol precursors nerolidol and geranyllinalool under the action of terpenoid synthase, and second, the oxidation of the nerolidol and geranyllinalool CYP genes to produce DMNT and TMTT [109] (Figure 2). Specifically, the ethylene groups on geranyllinalool and nerolidol are epoxidized to remove C2 and form the intermediate geranyl acetone (C13) or farnesyl acetone, which is then deacetylated to form DMNT (C11) or TMTT (C16).

For a long time after it was discovered in 1989 that the precursors of the terpene homologues DMNT and TMTT were nerol and geranyl linalool, many researchers explored the conversion of nerol and geranyllinalool into terpene homologues in plants. TMTT was detected in *Arabidopsis thaliana* after JA treatment. After *Arabidopsis thaliana* was treated with alamethicin (ALA, an elicitor), AtCYP82G1 expression was upregulated and co-expressed with the geranyllinalool synthase GES gene [109]. The protein expressed in *Saccharomyces cerevisiae* can react with nerolidol and geranyllinalool to form DMNT and TMTT, and it can compensate for the phenotype in which the mutant version of the gene cannot produce TMTT. It is therefore considered the key enzyme for the synthesis of terpene homologues. This gene belongs to the CYP82 family and exists only in the dicotyledons. DMNT and TMTT are synthesized by co-eliminating the polar head of the substrate and the C5 hydrogen atom of the allyl group. The encoded enzyme has low substrate specificity and can use nerol or geranyllinalool as a substrate. It can also use the (E)-nerolidol 3s isomer as a substrate, but it cannot use linalool, (Z)-nerolidol, (E,E,E)-geranyllinalool, (E,E)-farnesol, (E)-geranyl alcohol, or fully saturated analogs of the two substrates [109]. The oxidative cracking of (E)-nerolidol and (E,E)-geranyllinalool depends on the elimination of β-carbon atoms [58].

## 7. Research and Application Prospects

At present, chemical pesticides are mainly used to control pests in agricultural production. However, their extensive use results in a series of negative effects, such as environmental destruction, poorer crop quality, and pesticide residues. They also cause pests to develop resistance to pesticides, reducing their effectiveness over time. Terpenoids exist widely in all kinds of plants, and when plants are damaged, the released of these terpenoids can be induced, thereby attracting the natural enemies of pests to achieve indirect defense. The natural enemies of pests will evolve with the evolution of the pests themselves; therefore, in theory, the indirect defense strategy by which the natural enemies of pests are attracted cannot be overcome by pests and should persist over time. In recent years, this feature has also been used in the “push–pull” pest control strategy, which aims to make use of the sensory systems of insects, such as their senses of smell, vision, and taste, along with a series of behaviors such as feeding, courtship, and avoidance, to avoid or trap pests through corresponding plant resources, artificial simulation materials, or chemical synthetic substances [110,111]. This strategy has been used both domestically and internationally. Small-scale farmers in sub-Saharan Africa have adopted this strategy to manage pests. For example, the intercropping of cattle forage grass and corn provides the power of a “push” to expel corn borer-stemmed Noctuidae (*Helotrophaleu costigma*) and maize stem borers (*Chilo partellus*) from the corn fields [112]. (E)-β-farnesene (EβF) has also been applied to rice in China. It was found that EβF could control the population growth of rice pests by attracting their natural enemies, such as ladybugs [37]. By causing a reaction between a pseudo-substrate resembling the natural substrate and geraniene D synthase, a target product with a similar structure and similar properties, but with a more efficient biological function, can be produced [113]. Modifications of nerolidol and geranyllinalool are underway [114]. For example, researchers have begun replacing their methyl groups R′, R″ and R‴, or forming cyclization between their C6 and C11 or C15 positions, resulting in pseudo-substrates with similar structures. The natural cytochrome monooxygenase P450 in plants can react with pseudo-substrates to produce stable functional analogs of DMNT and TMTT, and these can be used in the field. In contrast, transgenic technology can also be used to improve crops so that they can release more resistance-related terpenoids and “pull” the natural enemies of pests, such as the natural enemy of the striped stemborer (*Chilo suppressalis*, *Cotesia chilonis*). Many studies have proven the feasibility of this strategy. For example, transgenic *Arabidopsis thaliana* can produce large amounts of nerol and DMNT and attract more predatory mites by transferring nerol synthase from strawberries into the mitochondria of *Arabidopsis* [115]. DMNT and TMTT transgenic rice plants are significantly more attractive to *C. chilonis* female wasps than wild type ZH11 plants [116]. To avoid energy waste in genetically modified crops, growers can consider using pest-feeding inducible promoters to drive these genes, making this strategy more efficient and feasible. Therefore, the use of terpene plant volatiles to encourage crop–pest–natural enemy tritrophic interactions in the field is an effective and sustainable method of pest control.

Many studies have shown that when pests damage plants, the expression of the terpene synthase genes in these plants will sharply increase [64,66,102,109], though so far there is very little research on the ways in which plants can induce the synthesis of terpene synthase. It is possible to excavate the relevant regulatory transcription factors to improve the synthesis regulatory networks of terpene homologues, thereby regulating the release of terpene homologues by controlling the expression levels of these upstream transcription factors, and thereby improving the ability of plants to resist pests.

## Figures and Tables

**Table 1:** Correspondence between terpenoid synthesis genes and transcription factors in different species.

Species	Terpene	Genes	TF	Reference
*Actinidia arguta*	Terpinolene	*AaTPS1*	*AaNAC*	[73]
*Arabidopsis thaliana*	(E)-β-caryophyllene	*AtTPS11*; *AtTPS21*	*AtMYC2*	[74]
*Artemisia annua*	Artemisinin	*AaGSW1*;	*AaWRKY1*; *AaERF1*; *AaERF2*; *AabZIP1*; *AaSPL2*	[75,76,77,78]
*Citrus sinensis*	(E)-geraniol	*CitTPS16*	*CitERF71*	[79]
*Gossypium arboreum*	Gossypol	*CAD1-A*	*GaWRKY 1*	[80]
*Medicago truncatula*	Saponin	*TSAR1*; *TSAR2*	*TSAR1*; *TSAR2*	[81]
*Mentha spicata*	α-Pinene; β-Pinene; eucalyptol; linalyl acetate; α-bergamotene; germacrene D; γ-muurolene; β-copaene; Limonene	*MsGPPS*; *MsNTT*	*MsMYB*; *MsYABBY5*	[82,83]
*Oryza sativa*	Phytoalexins	*OsDXS3*	*OsWRKY45*; *OsTGAP1*; *OsbZIP79*	[84,85,86]
*Panax quinquefolius* (*Arabidopsis thaliana*)	Ginsenoside	*AtHMGR*; *AtFPS2*; *AtSQS1*; *AtSQE2*	*PqWRKY1*	[87]
*Phalaenopsis bellina*	Geraniol; linanol	*PbGDPS*; *PbGDPS2*; *PbTPS5&7&9&10*	*PbbHLH4*; *PbbHLH6*	[88]
*Salvia miltiorrhiza*	Tanshinones	*SmCPS1*; *SmKSL1*; *SmCYP76AH1*	*SmWRKY1*; *SmWRKY2*; *SmMYB36*; *SmERF128*	[89,90,91,92]
*Solanum lycopersicum*	Carotenoid	*SlACS2*; *SIACS4*	*SINAC4*	[93]
*Taxus chinensis*	Taxol	*TcDBAT*	*TcWRKY1*	[94]
*Zea mays*	(E)-β-farnesene	*ZmTPS10*	*ZmEREB58*	[95]

## Data Availability

Not applicable.
